# The interaction of unfolding α-lactalbumin and malate dehydrogenase with the molecular chaperone αB-crystallin: a light and X-ray scattering investigation

**Published:** 2010-11-18

**Authors:** Justyn W. Regini, Heath Ecroyd, Sarah Meehan, Kristen Bremmell, Matthew J. Clarke, Donna Lammie, Tim Wess, John A. Carver

**Affiliations:** 1 School of Optometry and Vision Sciences, Cardiff University, Cardiff, UK; 2 School of Chemistry & Physics, The University of Adelaide, Adelaide, SA, Australia; 3 School of Biological Sciences, University of Wollongong, Wollongong, NSW, Australia; 4 Department of Chemistry, University of Cambridge, Cambridge, UK; 5 School of Pharmacy and Medical Sciences, The University of South Australia, Adelaide, SA, Australia

## Abstract

**Purpose:**

The molecular chaperone αB-crystallin is found in high concentrations in the lens and is present in all major body tissues. Its structure and the mechanism by which it protects its target protein from aggregating and precipitating are not known.

**Methods:**

Dynamic light scattering and X-ray solution scattering techniques were used to investigate structural features of the αB-crystallin oligomer when complexed with target proteins under mild stress conditions, i.e., reduction of α-lactalbumin at 37 °C and malate dehydrogenase when heated at 42 °C. In this investigation, the size, shape and particle distribution of the complexes were determined in real-time following the induction of stress.

**Results:**

Overall, it is observed that the mass distribution, hydrodynamic radius, and spherical shape of the αB-crystallin oligomer do not alter significantly when it complexes with its target protein.

**Conclusions:**

The data are consistent with the target protein being located in the outer protein shell of the αB-crystallin oligomer where it is readily accessible for possible refolding via the action of other molecular chaperones.

## Introduction

Small heat shock proteins (sHsps) are a diverse family of intracellular molecular chaperones that are found in all organisms [1,2]. In humans, they are present in many tissues at varying levels depending on the stage of development and the level of physiologic stress. The role of sHsps in cells is multi-faceted with their common theme of action being to interact with and stabilize partially folded states of other (target) proteins to prevent their aggregation and possible precipitation, for example under conditions of environmental stress such as elevated temperature, low pH and oxidation [2-5]. In vitro, sHsps prevent stress-induced aggregation of a variety of unrelated target proteins that undergo either disordered (amorphous) or ordered (amyloid fibril) forms of aggregation [2,6,7].

The principal eye lens protein, α-crystallin, is a sHsp that comprises two closely related subunits, αA- and αB-crystallin, each of which is ~20 kDa in mass. In the human lens, the two isoforms are co-expressed in a ratio of 3:1 αA-:αB-crystallin [8]. The two isoforms form a heterogeneous oligomeric species of average mass of approximately 800 kDa and 150 Å in diameter [8]. The lens contains a very high concentration of protein (up to 450 mg/ml in the nucleus or center of the lens) encased in very long fiber cells. α-Crystallin is the primary protein component of the lens and can approach 50% of the total dry weight of the lens [9]. α-Crystallin has two important functions in the lens. First, in a structural role, it assists in the maintenance of short-range order in the lens cytoplasm, ensuring proper refraction of light and maintenance of lens transparency [10]. Second, it acts as a molecular chaperone to maintain the solubility of the other classes of crystallin proteins, β- and γ-crystallin. It is also known to protect other non-crystallin lens proteins such as sorbital dehydrogenase from both thermal aggregation and enzyme inactivation [11]. There is no protein turnover in the center of the lens, meaning that the crystallin proteins have to be very long lived; α-crystallin is involved in minimising lens protein precipitation over decades, and thereby the prevention of lens opacification and cataract formation [12].

Outside the lens, αB-crystallin is also expressed at significant levels [13] where it has a key role as a molecular chaperone. For example, in addition to its role in the prevention of cataract, αB-crystallin is of interest extralenticularly because its expression is associated with many other protein misfolding disorders. Thus, αB-crystallin is found in significant levels in the brains of patients with Alzheimer's disease [14,15], Parkinson disease [16,17], in multiple sclerosis [18,19], and in the ischemic heart [20]. In vitro, αB-crystallin protects target proteins against reduction-induced precipitation [21,22], heat-induced aggregation [6,23], enzyme inactivation [24-26], and amyloid fibril formation [7].

The polydispersity of α-crystallin means that its assemblages can vary considerably in their number of subunits. Cryo-electron microscopic (cryoEM) studies of the αB-crystallin oligomer [27] show that it contains a spherical protein shell of 80 to 180 Å in diameter surrounding a central cavity measuring 30 to 100 Å in diameter, as well as a region on the protein surface that is highly dynamic which arises from the flexible COOH-terminal extensions [28,29]. The heterogeneous and dynamic nature of the α-crystallin oligomer has precluded crystal formation and thereby precise atomic structural resolution of the protein and specific details about its mechanism of chaperone action.

Previously, we used low-angle X-ray scattering from solutions of extracted bovine α-crystallin on its own and in the presence of β-crystallin to study structural changes in α-crystallin during chaperone action as a function of temperature [30,31]. The α-crystallin oligomer underwent extensive structural changes and became much larger at higher temperature, with a major transition at around 50 °C. We used the term ‘super aggregation’ to describe the enlargement of the α-crystallin oligomer with increasing temperature. Our results were consistent with earlier transmission electron microscopy, circular dichroism and non-denaturing gel electrophoresis studies of α-crystallin [32]. Interestingly, we also found that below 50 °C, a weak interaction occurred between α-crystallin and β-crystallin implying that the β-crystallin subunits may be transiently localized in the exterior fenestrations and/or the central cavity of the α-crystallin oligomer that have been described from electron microscopic studies of αB-crystallin. At higher temperatures (i.e., under conditions of partial unfolding of β-crystallin), the β-crystallin subunits were most likely bound to the surface of the α-crystallin oligomer [31]. Our recent neutron scattering studies on the interaction of the target protein γ_E_-crystallin with α-crystallin at 65 °C have provided further insight into the location of the target protein when interacting with α-crystallin under chaperone conditions. Under these relatively harsh stress conditions, the data are consistent with γ_E_-crystallin binding in the central cavity of the α-crystallin oligomer [33].

Our previous X-ray solution scattering experiments used extracted bovine α- and β-crystallins, both of which are comprised of several isoforms. While such experiments are relevant to the in vivo situation in the eye lens, they do not reflect of the situation in other tissues where only αB-crystallin is found. Furthermore, ascribing the observed experimental structural changes to particular species in such multi-component mixtures is difficult. Thus, the purpose of this study was to examine the interaction of recombinant αB-crystallin with the well characterized target proteins, α-lactalbumin (α-LA) and malate dehydrogenase (MDH). α-LA is a small monomeric milk protein (mass approximately 14 kDa) that has four disulphide bonds in its native state. When these bonds are reduced, α-LA forms an intermediately folded (molten globule) state that aggregates and precipitates out of solution [34-36]. The precipitation of α-LA can be prevented by a sufficient quantity of αB-crystallin [34-36]. α-LA is an attractive target protein to study as its folding pathway, and its various intermediate states, have been well characterized. There have also been a variety of biophysical studies undertaken on the interaction of reduced α-LA with αB-crystallin including our detailed real-time spectroscopic and biophysical investigations in which we showed that αB-crystallin interacts with reduced, partially folded, monomeric α-LA to prevent its aggregation and precipitation [34-36] During this interaction, αB-crystallin acts on the destabilized molten globule form of α-LA, which consequently retains some secondary structure within the complex formed with the chaperone protein [34-36]. Malate dehydrogenase (MDH) is an enzyme involved in the citric acid cycle that catalyzes the conversion of malate to oxaloacetate and exists as a dimeric or tetrameric enzyme comprised of identical subunits each of mass between 30 and 35 kDa [37]. Under mild thermal stress, MDH partially unfolds, aggregates and precipitates and therefore has been used as a target protein to investigate the chaperone activity of both α-crystallin and αB-crystallin [26,38].

Here we describe, for the first time, the use of X-ray solution scattering to investigate the complex formed between αB-crystallin and its target proteins α-LA and MDH. In addition, we have used dynamic light scattering (DLS) to characterize the size of the complex formed between αB-crystallin and α-LA. The X-ray scattering and DLS experiments enabled real-time measurements to be made of the structural alterations that occur when αB-crystallin interacts with reduced α-LA to prevent its aggregation and precipitation. Overall, the α-LA data presented herein are consistent with our previous studies [36] and the cryoEM studies of Stewart and coworkers [3,27,38]. We find that the size of the αB-crystallin oligomer is very similar to that determined from cryoEM measurements and that, in the complex formed between αB-crystallin and α-LA, the data are consistent with α-LA being located in the outer protein shell of the αB-crystallin oligomer. Similarly, from the light and X-ray scattering experiments of the interaction of thermally stressed MDH with αB-crystallin, we were able to monitor structural changes in real time of the proteins during chaperone interaction and conclude that a similar mode of interaction occurs as between reduced α-LA and αB-crystallin.

## Methods

The vector pET24d(+) (Novagen, Madison, WI) containing the gene for expression of human αB-crystallin was a gift from Professor W. Boelens (University of Nijmegen, Netherlands). Human recombinant αB-crystallin was expressed and purified as described previously [39]. Calcium-depleted bovine α-LA and MDH (mitochondrial from porcine heart) were purchased from Sigma (Gillingham, UK). All other chemicals were of the highest grade.

### Light scattering and dynamic light scattering monitoring of the chaperone action of αB-crystallin against reduced α-LA

Aggregation assays of αB-crystallin and α-LA were undertaken using methods outlined previously [22,35,36]. Briefly, α-LA (2 mg/ml) was dissolved in 50 mM phosphate buffer, 100 mM NaCl, 2.5 mM EDTA at pH 7.2, and incubated at 37 °C. Dithiothreitol (DTT, 20 mM) was added to the solution to induce reduction, unfolding and aggregation of the protein. Light scattering at 340 nm was monitored over time using a Cary 5000 UV/Vis/NIR spectrophotometer (Varian, Melbourne, Australia). The DLS measurements were recorded using a Zetasizer Nano ZS (Malvern Instruments, Worcestershire, UK). Accumulation times for each sample were determined automatically, and the temperature was controlled at 37.0 °C±0.1. The inbuilt software used the correlation function to calculate the z-average (intensity mean) hydrodynamic diameter (*D_H_*) and the translational diffusional coefficient (*D_T_*). The distributions of hydrodynamic diameters were calculated according to the Stokes-Einstein equation:

$$D_{H}=kT/3\pi \eta D_{T}\text{ Equation 1}$$

where *k* is the Boltzmann constant, *T* is the absolute temperature, and *η* is the solvent viscosity. The diameter measurements were converted to measurements of the hydrodynamic radius (*R_H_*) for ease of comparison with the radius of gyration (*Rg*) measurements determined from the X-ray solution scattering data.

### Light scattering monitoring of the chaperone action of αB-crystallin against heat-stressed MDH

Solutions containing 50 mM phosphate buffer, 100 mM NaCl, 2.5 mM EDTA at pH 7.5 were used. Two solutions were studied; the first contained 0.25 mg/ml MDH only, and the second contained 0.25 mg/ml MDH and 0.1 mg/ml αB-crystallin, i.e., a 2.5:1.0 w:w ratio of MDH: αB-crystallin. These concentrations are consistent with previous MDH and α-crystallin chaperone studies [26,38]. The solutions were placed in 1 ml quartz cuvettes pre-heated to 42 °C in a Digilab Hitachi U-2800 spectrophotometer for 112 min and light scattering was measured at a wavelength of 360 nm The solutions were left for 5 min to equilibrate at 42 °C, as measured by a thermocouple (Cormark electronics Ltd., Littlehampton, UK).

### X-ray solution scattering measurements

Low-angle X-ray solution scattering experiments were conducted at two synchrotrons. Initially, beamline X33 at the European Molecular Biology Laboratory (EMBL) of the Deutsches Elektronen Synchrotron (DESY), Hamburg, Germany, was used. The wavelength was λ=1.5 Å, with a sample to detector distance of 2.4 m. covering a scattering range of 0.09 nm^−1^<*q*<4.98 nm^−1^ (*q=4π sinθ/λ*). The detector was calibrated using the in-house EMBL software. Experiments were also undertaken at Station 2.1 of the Daresbury Synchrotron Radiation Source, Warrington, UK. The camera length was 5.25 m, with a corresponding scattering range of 0.00056 nm^−1^<*q*<9.72 nm^−1^. The wavelength was λ=1.5 Å and the detector was calibrated using hydrated rat tail tendon.

As with the light scattering assays, all experiments were performed in 50 mM phosphate buffer, 100 mM NaCl, 2.5 mM EDTA at pH 7.2. After an initial X-ray exposure, DTT was added (to a final concentration of 20 mM) to the solutions containing α-LA alone (2 mg/ml) and α-LA combined with αB-crystallin, and the samples were monitored with time. Samples containing mixtures of αB-crystallin and α-LA were prepared at 1:0, 1:1, and 1:10 w:w ratios, giving protein concentrations of 2 mg/ml αB-crystallin:0 mg/ml α-LA, 2 mg/ml αB-crystallin:2 mg/ml α-LA, and 2 mg/ml αB-crystallin:0.2 mg/ml α-LA, respectively. From our previous X-ray solution scattering studies [30,31], we found that the optimum signal to noise ratio of α-crystallin and target protein is achieved with concentrations between 2 and 4 mg/ml. An initial X-ray exposure was acquired before the addition of DTT, then every 8 min for a total time of 136 min. The temperature was maintained at 37 °C.

For the MDH studies, X-ray exposures were taken every 8 min for a total of 112 min for solutions containing 2 mg/ml of MDH in the absence and presence of 2 mg/ml αB-crystallin in the same buffer as per the light scattering experiments. The temperature was maintained at 42 °C.

For both sets of experiments, the exposure times were 60 s each, and the temperature was kept constant with a thermostated circulating bath pumping fluid through the sample holder.

The X-ray data were recorded and analyzed using the PRIMUS software package [40]. In all cases, sector integrations were performed with the origin at the position of the direct beam masked by the backstop. This method improves the signal-to-noise ratio at higher scattering angles, which is of particular significance for weakly scattering samples such as proteins in solution. All intensity profiles were corrected for background scattering. In all experiments, Guinier analysis was used to determine the average radius of gyration (*Rg*) of the protein aggregates as a function of temperature following our earlier work [30,31]. The *Rg* value is derived from the Guinier region of the solution scattering X-ray intensity profiles. Such regions were used to fit the Guinier approximation Equation [41].

$$I\;=\;I_{0}\text{ exp}(-{\text{4p}}^{\text{2}}\;S^{\text{2 }}Rg^{\text{2}}/\text{3})\;\;\;\;\;\;\;\;\;\;\;\;\;\;\;\;\;\;\;\;\;\text{ Equation 2}$$

Where, *I* is the scattered intensity and *I*_0_ the forward scattering intensity and *S* is inverse space. The *Rg* value is the root mean square distance of the electrons of the molecules in solution from the centers of their electronic masses and, therefore, is a measure of the overall size of the molecules. All *Rg* values were found using PRIMUS software and satisfied the Guinier condition of *Rg q*≤1.3. The ratio of *Rg* and *R_H_*, the hydrodynamic radius, leads to the dimensionless parameter ρ, i.e.

$$\rho \;=\;Rg\;/\;R_{H}\;\;\;\;\;\;\;\;\;\;\;\;\;\;\;\;\;\text{ Equation 3}$$

which is strongly dependent on the shape of the molecule [42].

## Results

### αB-crystallin and α-LA

#### Light scattering experiments

**Figure 1:**
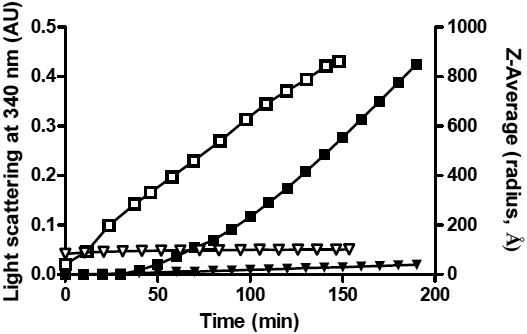
Monitoring the DTT-induced amorphous aggregation of α-LA by light scattering at 340 nm (solid symbols) and dynamic light scattering (open symbols). In both experiments α-LA (2 mg/ml) was incubated at 37 °C in 50 mM phosphate buffer, 100 mM NaCl, 2.5 mM EDTA at pH 7.2 with 20 mM DTT, in the absence (squares) or presence (triangles) of αB-crystallin (1:1 w:w ratio of α-LA: αB-crystallin). The change in light scattering at 340 nm is shown on the left y-axis and the Z-average hydrodynamic radius of particles (Z-average radius, Å) measured by DLS over time is shown on the right y-axis.

When reduced with DTT, α-LA (2 mg/ml) at 37 °C underwent amorphous aggregation and precipitation as monitored by light scattering at 340 nm (Figure 1). There was a lag phase of ~30 min followed by an exponential increase in light scattering over the remaining 200 min. The addition of αB-crystallin at a 1:1 w:w ratio of α-LA: αB-crystallin completely suppressed this increase in light scattering indicating that the chaperone prevents the precipitation of α-LA. Under these conditions, very similar behavior was observed in previous studies of the interaction between these two protein [22,43]. αB-Crystallin interacts in a chaperone manner with partially unfolded, reduced and monomeric α-lactalbumin forming a complex with it and thereby preventing its large-scale aggregation and precipitation. Our previous work has shown that complexation between the two proteins is established very quickly following the addition of DTT [34-36].

**Figure 2:**
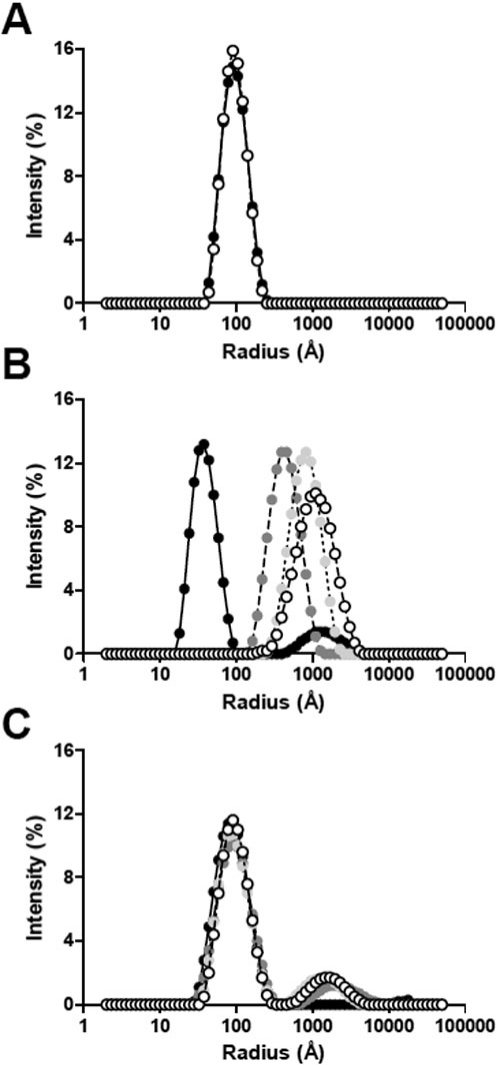
Dynamic light scattering measurements of the changes in the distribution of particle sizes (hydrodynamic radii, *R_H_*) during the DTT-induced aggregation of α-LA at 37 °C in the absence and presence of αB-crystallin over time. The plots show the relative light scattering intensities (%) of particles of increasing *R_H_* (Å) for **A** αB-crystallin + DTT alone, **B** DTT-reduced α-LA alone, and **C** DTT-reduced α-LA in the presence of αB-crystallin (1:1 w:w ratio of α-LA: αB-crystallin). Particle distributions are shown for 0 min (black), 50 min (dark gray), 100 min (light gray) and 150 min (white) following addition of DTT. The *R_H_* values used in deriving ρ (see Discussion) are based on the weighted mean of the major peaks in **A** and **C**.

As monitored by DLS, the Z-average hydrodynamic radius of all α-LA particles in solution, when incubated in the absence of αB-crystallin increased immediately following the addition of DTT (i.e., there was no discernable lag phase; Figure 1). The lack of a lag phase in the DLS measurements compared to monitoring α-LA aggregation by light scattering at 340 nm (Figure 1) is reflective of the ability of DLS to detect very small changes in particle size that do not result in detectable levels of light scattering. As shown in Figure 2, the DLS measurements also allow for the determination of the *R_H_* value of individual peaks that contribute to the Z-average hydrodynamic radius, as well as the heterogeneity of each peak (as determined from the width of each peak at its base). The Z-average hydrodynamic radius of α-LA particles present in solution increased over the time-course of the assay, from ~40 Å (after 3 min, the first reading after DTT was added) to 860 Å by the end of the assay (150 min; Figure 1). When the individual components that contribute to this Z-average radius were examined, this increase was observed to be due to the formation of increasingly large and polydisperse mixtures of α-LA aggregates with *R_H_* values>1000 Å (Figure 2B).

The Z-average hydrodynamic radius of αB-crystallin alone in solution was 85±18 Å. The αB-crystallin oligomers were found to range in size from a *R_H_* value of ~50 Å to 190 Å (based on the width of the peak at its base, see Figure 2A). When αB-crystallin was added to DTT-treated α-LA at a 1:1 w:w ratio, i.e., conditions under which large scale aggregation of α-LA is prevented (Figure 1), there was a small increase in the Z-average radius of the solution from 82 Å to 100 Å over the first 60 min which then remained constant for the remaining 90 min of the assay (Figure 1). Figure 2C, which shows the size of the particles in solution, indicates that at 50 min there were two predominant species present in the 1:1 mixture of α-LA: αB-crystallin, i.e., a smaller sized aggregate (*R_H_* ~100 Å with a peak width from 40 Å to 300 Å) and a minor, larger-sized aggregate (*R_H_*>750 Å). In their studies of the interaction of α-LA and α-crystallin under reduction conditions, Bettleheim et al. [44], also observed these two different populations and demonstrated, using size exclusion HPLC and SDS–PAGE, that the peak at ~100 Å represents the complex formed between αB-crystallin and α-LA and that the larger peak (*R_H_*>750 Å) is attributable to aggregated α-LA alone. Interestingly, the investigations here show that when αB-crystallin was present, the larger-sized aggregate of α-LA remained of similar size for the remainder of the assay. Moreover, the amount of this α-LA aggregate, as a proportion of the total number of particles in solution, remained small (i.e., ~14%; Figure 2C) unlike when the chaperone was not present (Figure 2B), where the large aggregates (*R_H_*>1000 Å) represented most (i.e., >95%) of the particles in solution.

#### X-ray scattering experiments

**Figure 3:**
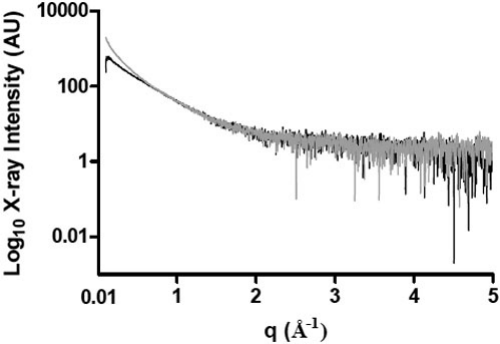
The X-ray intensity profiles plotted against the inverse space (*q*) of α-LA at 37 °C, 32 min (black) and 72 min (gray) after the addition of DTT.

X-ray scattering experiments of αB-crystallin and α-LA solutions on their own and initially without the addition of DTT, each at 2 mg/ml, gave *Rg* values of 58.2 (±0.1) Å and 22.4 (±0.2) Å respectively. The large difference in *Rg* values between the two proteins is consistent with the much greater size of the αB-crystallin oligomer (~650 kDa in mass) compared to the α-LA monomer (~14 kDa). Figure 3 shows the X-ray intensity scattering profiles of reduced α-LA, 32 and 72 min after the addition of DTT. To highlight the scattering features at higher angles, the intensity is plotted on a logarithmic scale. The increase in the X-ray intensity at low angles with time is an indication of aggregation [30,31]. From Figure 3, it is apparent that in the region beyond q=2, the X-ray data become noisy and imprecise. In monodisperse systems, this is the region where information is obtained from the particle shape and Fourier transform and a shape reconstruction may be performed. One obvious result of the large amount of noise in the data in this region of Figure 3 is that Fourier transforms from different-sized aggregating particles are overlaid and smear out the data meaning that shape reconstruction is not feasible, as is also the case in the polydisperse αB-crystallin and α-LA mixtures under reducing conditions (data not shown).

**Figure 4:**
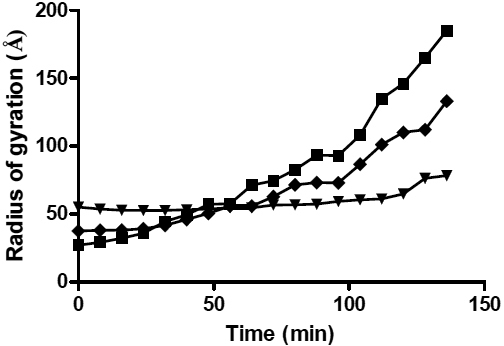
The variation in the radius of gyration (*Rg*) with time after the addition of 20 mM DTT to α-LA (squares), and in the presence of αB-crystallin at a 1:1 (triangles) and 1:10 (diamonds) w:w ratios to α-LA. The standard deviations associated with these data, which represent the standard deviation from the line of best fit in the Guinier region, are too small to be distinguished in this plot. The *Rg* values used in deriving ρ (see Discussion) are taken from the final *Rg* values in this plot.

The initial *Rg* value of reduced α-LA (from data acquired as soon as possible after addition of DTT) is 26.9 (±0.2) Å which exhibits a steady increase with time (particularly after 50 min associated with the formation of large light scattering aggregates, Figure 1) to a value of 108.0 (±0.2) Å at 104 min and 185.0 (±0.6) Å after 136 min (Figure 4). In contrast, the solution containing αB-crystallin and reduced α-LA at a 1:1 w:w ratio showed only a slight increase in the *Rg* value from 55.1 (±0.1) Å initially to 60.4 (±0.1) Å 104 min after addition of DTT (Figure 4), through to a value of 78.2 (±1.1) Å at 136 min after addition of DTT, i.e., significantly lower than that measured for α-LA in the absence of αB-crystallin. From our previous work [34,36], it is well established that under these conditions and time frame, αB-crystallin is an efficient chaperone for reduced α-LA (see Figure 1) whereby it interacts and complexes with α-LA to prevent it from aggregating. Thus, these *Rg* values correspond to the complex formed between the two proteins and are not simply due to averaging of the *Rg* values of the two components present. Evidence for this is that if the *Rg* values simply reflected an averaging of the two components, they would increase greatly over time due to the unfolding and aggregation of the reduced α-LA.

The initial *Rg* value of the solution containing a 1:10 w:w ratio of the αB-crystallin and α-LA mixture was 37.4 (±0.2) Å, which, because of the excess of α-LA, was much lower than the initial *Rg* value (55.1 [±0.1] Å) of the 1:1 w:w ratio (Figure 4). The *Rg* values for the 1:10 mixture then increased significantly with time following addition of DTT, reaching a value of 133.0 (±1.4) Å after 136 min, which is consistent with the inability of αB-crystallin to function efficiently as a chaperone for reduced α-LA at this sub-stoichiometric ratio [36]. As a result, significant aggregation and precipitation of reduced α-LA occurs, as we have previously demonstrated [34,36].

**Figure 5:**
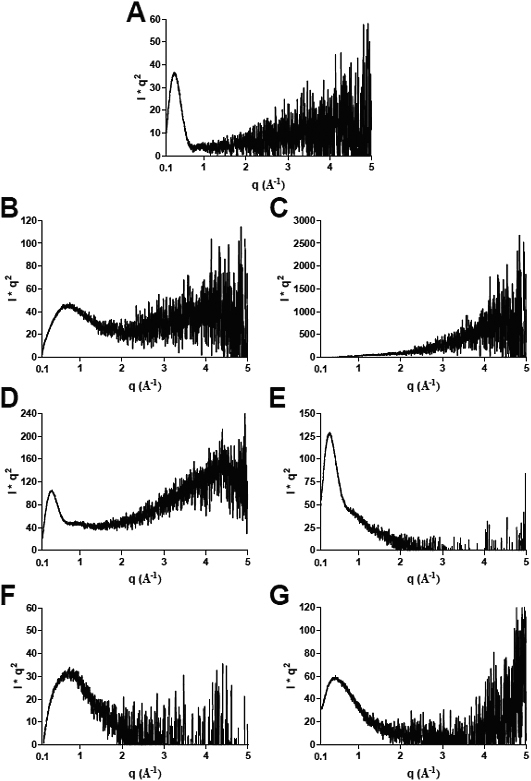
X-ray intensity profiles plotted against I*q^2^. Kraty plots are shown for **A** αB-crystallin alone, α-LA + DTT at **B** 0 min, and **C** 136 min, αB-crystallin plus α-LA at a 1:1 w:w ratio at **D** 0 min and **E** 136 min, and αB-crystallin plus α-LA at a 1:10 w:w ratio at **F** 0 min and **G** 136 min.

Figure 5 shows Kratky plots at the start and end of the X-ray solution scattering experiments for αB-crystallin alone (Figure 5A), α-LA plus DTT (Figure 5B,C), αB-crystallin plus α-LA at the 1:1 w:w ratio (Figure 5D,E) and αB-crystallin plus α-LA at the 1:10 w:w ratio (Figure 5F,G). For these Krakty plots, the X-ray intensity scattering profiles (Figure 3) are plotted as *Ixq^2^* against *q*, where *I* is the scattered intensity and *q=4π sinθ/λ*. As can be seen in Figure 5A, αB-crystallin alone shows a peak centered around *q*=0.3. The presence of such a peak indicates that the majority of particles in solution are globular [45] and therefore approximately spherical in shape. From Figure 5B,C, it is apparent that α-LA loses its globular structure upon unfolding, aggregation and precipitation associated with the reduction of its four disulfide bonds and the adoption of a molten globule conformation [34,36]. As a result, the peak centered at *q*=0.7 at 0 min in its Kratky plot is lost by 136 min after addition of DTT. By contrast, the presence of a peak at *q*=0.3 in the plots at 136 min for the αB-crystallin plus α-LA mixtures at both the 1:1 and 1:10 w:w ratios (Figure 5E,G) clearly indicates that, under both conditions, the αB-crystallin/α-LA complex has a spherical shape.

### αB-crystallin and ΜDΗ

#### Light scattering experiments

**Figure 6:**
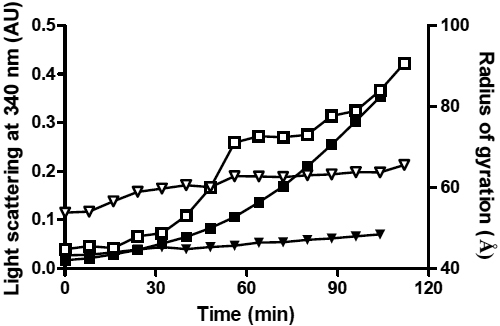
Monitoring the thermally-induced amorphous aggregation of MDH by light scattering at 360 nm (solid symbols) and SAXS (open symbols). In both experiments a 2 mg/ml solution of MDH was incubated at 42 °C in 50 mM phosphate buffer, 100 mM NaCl, 2.5 mM EDTA at pH 7.5 in the absence (squares) or presence (triangles) of αB-crystallin (2.5:1.0 w:w ratio of MDH and αB-crystallin for the light scattering experiments and a 1.0:1.0 w:w ratio of MDH and αB-crystallin for the SAXS experiments). The change in light scattering at 360 nm is shown on the left y-axis and the radius of gyration of the samples over time is shown on the right y-axis. The standard deviations associated with the SAXS data, which represent the standard deviation from the line of best fit in the Guinier region, are too small to be distinguished in this plot.

The chaperone activity of αB-crystallin in solution under mild heating conditions at 42 °C was investigated with MDH as the target protein. Figure 6 shows that the light scattering of MDH with time at 42 °C is exponential following a lag period of 15 min. By contrast, the light scattering of a 2.5:1.0 w:w solution of MDH:αB-crystallin shows only a very minimal increase over the same time period, demonstrating that αB-crystallin acts as a molecular chaperone to prevent the temperature-induced, partial unfolding and aggregation of MDH.

#### X-ray scattering experiments

Figure 6 also shows the *Rg* value for MDH alone and for a 1:1 w:w mixture with αB-crystallin during incubation at 42 °C. The initial *Rg* value of ΜDΗ is 47.7 (±0.3) Å which exhibits a steady increase with time to a value of 59.9 (±0.4) Å at 48 min. The *Rg* value then increases rapidly to 71.1 (±0.5) Å at 56 min, After this, the *Rg* value increases further to 83.9 (±0.8) Å at 104 min. The initial *Rg* value of a 1:1 w:w mixture of MDH and αB-crystallin is 53.8 (±0.1) Å. As with the mixture of α-LA and αB-crystallin, this value is lower than that for αB-crystallin alone (58.2 [±0.1] Å), which is explained by the smaller MDH molecules causing a reduction in the average *Rg* value for both proteins in solution. At the initial time point, it is unlikely that the two proteins strongly interact as the MDH molecules will not have unfolded to any significant degree. After a sufficient period of time under thermal stress, MDH and αB-crystallin form a complex [38]. During the first 48 min at 42 °C, the *Rg* value of the mixture increases to 60.1 (±0. 3) Å and then more slowly to a value of 65.6 (±0.4) Å after 112 min.

## Discussion

αB-Crystallin is a member of the sHsp family that shares a conserved approximately 90 amino acid ‘α-crystallin’ domain and acts as a molecular chaperone protein by preventing the stress-induced aggregation and precipitation of target proteins. Crystallographic structures of two non-metazoan sHsps are available [1,46] along with the structure of a sHsp from a flatworm, Tsp36 [47]. Recently, the crystal structure of the mammalian ‘α-crystallin’ domain has been solved [48]. Several structural similarities exist between these sHsps, most notably for the two non-metaozoan sHsps, which are both approximately spherical aggregates with a large central cavity. The αB-crystallin oligomer also has a cavity at its center, as determined by EM studies [27,49,50], and as was predicted by simple consideration of mass distribution in the aggregate [51]. The conclusions from the cryoEM studies agree very well with those derived from the solution-based DLS and X-ray scattering data presented herein for the αB-crystallin oligomer. The mass of the αB-crystallin monomer is ~20 kDa and the cryoEM data were acquired on samples which contained αB-crystallin oligomers of mass around 650 kDa, i.e., comprising approximately 32 subunits [49]. The cryoEM data indicate that the diameter of the αB-crystallin oligomer is 147 (±28) Å, i.e., a radius of 74 (±14) Å [49]. In agreement with these values, from our DLS studies (Figure 2), we found that αB-crystallin has a Z-average hydrodynamic radius (*R_H_*) of 85±18 Å, which is also very similar to that previously reported for the α-crystallin oligomer (comprising both the αA- and αB-crystallin subunits) as measured by DLS [44]. Thus, these experimental techniques all gave very similar results with respect to the size of αB-crystallin oligomer.

The DLS and X-ray solution scattering data for reduced α-LA and its 1:1 w:w mixture with αB-crystallin imply that αB-crystallin interacts with destabilized, partially unfolded α-LA molecules early on in the latter’s aggregation (off-folding) pathway, i.e., well before large scale aggregation occurs for reduced α-LA. These findings support our previous conclusions determined from NMR studies of the interaction of these two proteins [34-36]. Our present data confirm that the interaction between the two proteins is established very quickly following addition of DTT since there is no significant change in the *Rg* and *R_H_* values with time from those obtained immediately after the addition of the reducing agent. For example, the DLS measurements indicate that reduced α-LA and αB-crystallin form a stable complex (of *R_H_* ~100 Å) within the dead time of the experiment (~5 min), and this prevents the increase in α-LA aggregate size that leads to precipitation when the chaperone is absent. Bettleheim et al. [44] reported similar results for the interaction of α-crystallin with reduced α-LA. In addition, in the presence of αB-crystallin, the larger α-LA aggregate (*R_H_*>750 Å) does not continue to grow in size, as occurred when the chaperone was absent (compare Figure 2B and Figure 2C), and it constitutes only a small percentage of the total number of particles in solution. These large particles are most likely too few in number to be detected by light scattering at 340 nm (due to its decreased sensitivity compared to DLS) and therefore a change in light scattering is not observed when α-LA is incubated in the presence of αB-crystallin. Higher concentrations of α-crystallin completely suppress the formation of this aggregated form of α-LA [44,52].

Other spectroscopic and biophysical studies on this system have also come to the conclusion that αB-crystallin acts early on to prevent the aggregation of destabilized α-LA molecules [34-36]. The X-ray solution scattering data for a 1:10 w:w ratio mixture of αB-crystallin:α-LA show that time-dependent aggregation of this mixture (as monitored by the change in *Rg* values) is only slightly inhibited compared to the situation with α-LA on its own where large-scale aggregation occurred. By contrast, time-dependent aggregation was almost completely absent at a 1:1 w:w ratio of the two proteins (Figure 1, Figure 2, and Figure 4). The rationale for these observations is that at a sub-stiochiometric 1:10 w:w ratio, αB-crystallin is not capable of completely suppressing reduced α-LA aggregation whereas it does so very effectively at a 1:1 w:w ratio [36].

Interestingly, our DLS studies showed that the size of the αB-crystallin-α-LA complex (i.e., a *R_H_* value of 100±22.5 Å) was similar to that of the αB-crystallin oligomer alone (a *R_H_* value of 85±18 Å) although the target protein-chaperone complex was more heterogeneous (compare Figure 2A and Figure 2C). In support of our DLS measurements, cryoEM images of the αB-crystallin-α−LA complex also show that it is comparable in size to the αB-crystallin oligomer (see Figure 7 in Haley et al. [53] and Figure 2 in Horwitz [3]), i.e., the radius of the αB-crystallin-α-LA complex was found to have a range of 65 to 100 Å (P. Stewart, Vanderbilt University, Nashville, TN personal communication) compared to that of the αB-crystallin oligomer itself of 40 to 90 Å [27,53]. Horwitz et al. [39],  also found no significant difference in the size of the αB-crystallin-α−LA complex (compared to the αB-crystallin oligomer alone) by gel filtration chromatography. By contrast, at the end of the X-ray scattering experiment we found that the 1:1 w:w mixture of reduced α-LA and αB-crystallin had a *Rg* value of 78.2 (±1.1) Å (compared to αB-crystallin alone (58.2 [±0.1] Å). In agreement with the latter value, Skouri-Panet et al. [54] used SAXS to examine the temperature and pressure-dependent changes in the structure of sHsps and found that a 3.7 mg/ml solution of αB-crystallin had a *Rg* value of 61 Å at 23 °C, in a solution containing a phosphate buffer at pH 6.8. On the other hand Spinozzi et al. [55] found the *Rg* value to be 52 Å in a solution containing a TRIS buffer. The slight variation in *Rg* values between this study and ours most likely arises from the different buffer conditions used since the size of the αB-crystallin oligomer is dependent on solvent conditions [56].

Intuitively, one would expect that an association between the two proteins during sHsp chaperone action would lead to the formation of a complex with an increased size, yet these data indicate that, while being more polydisperse, there is no significant increase in the size of the αB-crystallin-α-LA complex compared to the αB-crystallin oligomer alone, but there is an increase in the *Rg* value of the complex. To rationalise the DLS, cryoEM and X-ray solution scattering data, one must consider the parameter ρ, which relates the radius of gyration (*Rg*) with the hydrodynamic radius (*R_H_*; see Equation 3) and describes the distribution of mass and shape of the molecule. The calculated standard deviation of ρ is 22% due to the polydispersity of the αB-crystallin oligomer and the αB-crystallin-α−LA complex and therefore the range in *R_H_* values of these particles in solution. Based on the DLS and X-ray solution scattering data at 150 min, the ρ value for αB-crystallin alone is 0.68±0.14 (i.e., *Rg*=58.2±0.1 Å and *R_H_*=85±18 Å) and for the αB-crystallin-α−LA complex, ρ is 0.78±0.18 (i.e., *Rg*=78.2±1.1 Å and *R_H_*=100±22.5 Å). Thus, due to the heterogeneity of the two systems, the parameter ρ is the same and corresponds to a value approximating that of a solid sphere (i.e., ρ_solid sphere_ ≈0.76, ρ_hollow sphere_ ≈1.0) [42,57].

In other words, the distribution, size and shape of the αB-crystallin oligomer are very similar whether it is has bound target protein or not, since the hydrodynamic radius (*R_H_*, Figure 1 and Figure 2) and spherical shape (ρ value and Kratky plots, Figure 5) are not significantly altered upon complex formation between αB-crystallin and α-LA. Our finding that the globular (spherical) shape of αB-crystallin oligomer is retained following formation of a complex with α-LA (see Figure 5) is consistent with previous cryo-EM studies [53]. During chaperone action, this most likely arises from positioning the reduced α-LA molecules on the surface of the αB-crystallin aggregate [3,39,53] i.e., within the oligomer’s protein shell. The partially folded α-LA molecules could be located in the fenestrations that are on the surface of the αB-crystallin oligomer [31] and the non-mammalian sHsp oligomers [1,46,57]. As a result, the target protein would be readily accessible for refolding via the action of other molecular chaperones (e.g., Hsp70), in a process that requires ATP hydrolysis, when cellular conditions allow [58]. The dynamic, flexible and malleable nature of the αB-crystallin oligomer, particularly on its surface where the flexible COOH-terminal extensions are located [28,29,51] would facilitate the incorporation of the target protein (in this case α-LA) within its outer protein shell [5,59]. Indeed, our NMR studies show that the flexibility of the COOH-terminal extension is altered significantly upon binding of α-LA [60] implying that the extensions and bound α-LA molecules are localized nearby to each other. Stengel et al. [61] have recently used sophisticated mass spectrometry methods to investigate the oligomeric states of a non-mammalian sHsp, pea Hsp18.1, when interacting with a target protein, luciferase, under mild temperature stress. Unlike αB-crystallin, Hsp18.1 adopts a well defined oligomer (a 12-mer) in the absence of target protein at room temperature. Upon chaperone interaction with luciferase, however, Hsp18.1 forms a highly heterogeneous range of complexes containing different stoichiometries of luciferase and Hsp18.1. It is proposed that this temperature-induced heterogeneity of Hsp18.1 facilitates its interaction with a range of target proteins and also enables the target proteins to be readily accessible for refolding upon complexation. For αB-crystallin, the polydispersity is already present at physiologic temperatures which enables it to readily interact with target proteins in the absence of temperature stress. As a result, αB-crystallin is ‘primed’ for interaction with a diversity of target proteins. Indeed, X-ray crystallography has recently revealed that the polydispersity of αB-crystallin is facilitated by the presence of a nine amino acid palindromic sequence centered around P160 of the COOH-terminal region that participates in inter-subunit interactions via alignment in both directions of its sequence in both directions [62].

Similar results were observed in comparing the X-ray scattering results (*Rg* values versus time) for the two target proteins (α-LA and MDH) in the presence of αB-crystallin (Figure 4 and Figure 6, respectively). Thus, the interaction between the target proteins and αB-crystallin under different mild stress conditions (reduction at 37 °C and heating at 42 °C) leads to a stable complex that has a *Rg* value that varies little with time and is not significantly different to that of the αB-crystallin oligomer on its own. It is concluded that the arguments presented above for the interaction of α-LA with αB-crystallin also apply for the interaction of MDH with αB-crystallin.

Using small-angle neutron scattering (SANS) experiments in conjunction with isotopic substitution and contrast matching techniques, we recently investigated the interaction of a lens target protein, γ_E_-crystallin, with bovine α-crystallin, under harsh thermal stress, i.e., 65 °C [33]. Under these conditions, we concluded that at γE-crystallin is located within the central cavity of the α-crystallin oligomer. As discussed above, 65 °C is well above the temperature at which α-crystallin undergoes a major rearrangement of its secondary, tertiary and quaternary structures which, coupled with the protein’s inherent dynamism and its porous nature due to the ‘fenestrations’ on its surface [27,30,49,50], would facilitate ready access of target proteins to the central cavity of the α-crystallin oligomer. Thus, α-crystallin may have different protective chaperone mechanisms depending on the stress conditions, i.e., the target protein can either bind within the central cavity or to the surface of the outer shell depending on external environmental factors, including the size of the target protein and whether the target protein is aggregating amorphously or to form amyloid fibrils, the pH, the type of stress (e.g., reduction or elevated temperature), the rate of target protein aggregation and the temperature. Indeed, our studies have shown the importance of these factors in determining the efficiency of chaperone action of αB-crystallin against target proteins [23,59,63,64].

## References

[1] van Montfort RLM Basha E Friedrich KL Slingsby C Vierling E Crystal structure and assembly of a eukaryotic small heat shock protein. Nat Struct Biol 2001 8 1025 30 11702068 10.1038/nsb722

[2] Treweek TM Morris AM Carver JA Intracellular protein unfolding and aggregation: The role of small heat-shock chaperone proteins. Aust J Chem 2003 56 357 67

[3] Horwitz J Alpha-crystallin. Exp Eye Res 2003 76 145 53 12565801 10.1016/s0014-4835(02)00278-6

[4] Derham BK Harding JJ Alpha-crystallin as a molecular chaperone. Prog Retin Eye Res 1999 18 463 509 10217480 10.1016/s1350-9462(98)00030-5

[5] Carver JA Rekas A Thorn DC Wilson MR Small heat-shock proteins and clusterin: intra- and extracellular molecular chaperones with a common mechanism of action and function? IUBMB Life 2003 55 661 8 14769002 10.1080/15216540310001640498

[6] Horwitz J Alpha-crystallin can function as a molecular chaperone. Proc Natl Acad Sci USA 1992 89 10449 53 1438232 10.1073/pnas.89.21.10449 PMC50356

[7] Ecroyd H Carver JA Crystallin proteins and amyloid fibrils. Cell Mol Life Sci 2009 66 62 81 18810322 10.1007/s00018-008-8327-4 PMC11131532

[8] Harding JJ. Cataract: Biochemistry, epidemiology and pharmacology. Chapman and Hall: London; 1991.

[9] de Jong WW. Molecular and Cellular Biology of the Eye Lens, New York: Wiley Interscience; 1981.

[10] Delaye M Tardieu A Short-range order of crystallin proteins accounts for eye lens transparency. Nature 1983 302 415 7 6835373 10.1038/302415a0

[11] Marini I Moschini R Del Corso A Mura U Alpha-crystallin: an ATP-independent complete molecular chaperone toward sorbitol dehydrogenase. Cell Mol Life Sci 2005 62 599 605 15747064 10.1007/s00018-005-4474-z PMC11365903

[12] Boyle D Takemoto L Characterization of the alpha-gamma and alpha-beta complex: evidence for an in vivo functional role of alpha-crystallin as a molecular chaperone. Exp Eye Res 1994 58 9 15 8157104 10.1006/exer.1994.1190

[13] Bhat SP Nagineni CN alpha B subunit of lens-specific protein alpha-crystallin is present in other ocular and non-ocular tissues. Biochem Biophys Res Commun 1989 158 319 25 2912453 10.1016/s0006-291x(89)80215-3

[14] Renkawek K Voorter CE Bosman GJ van Workum FP de Jong WW Expression of alpha B-crystallin in Alzheimer's disease. Acta Neuropathol 1994 87 155 60 8171966 10.1007/BF00296185

[15] Shinohara H Inaguma Y Goto S Inagaki T Kato K Alpha B crystallin and HSP28 are enhanced in the cerebral cortex of patients with Alzheimer's disease. J Neurol Sci 1993 119 203 8 8277336 10.1016/0022-510x(93)90135-l

[16] Lowe J McDermott H Pike I Spendlove I Landon M Mayer RJ alpha B crystallin expression in non-lenticular tissues and selective presence in ubiquitinated inclusion bodies in human disease. J Pathol 1992 166 61 8 1311375 10.1002/path.1711660110

[17] Trojanowski JQ Goedert M Iwatsubo T Lee VM Fatal attractions: abnormal protein aggregation and neuron death in Parkinson's disease and Lewy body dementia. Cell Death Differ 1998 5 832 7 10203692 10.1038/sj.cdd.4400432

[18] van Noort JM vSAC, Bajramovic JJ., el Ouagmiri M, Polman CH, Lassmann H, Ravid R, The small heat-shock protein alphaB-crystallin as candidate autoantigen in multiple sclerosis. Nature 1995 375 798 801 7596414 10.1038/375798a0

[19] Ousman SS Tomooka BH van Noort JM Wawrousek EF O'Connor KC Hafler DA Sobel RA Robinson WH Steinman L Protective and therapeutic role for alphaB-crystallin in autoimmune demyelination. Nature 2007 448 474 9 17568699 10.1038/nature05935

[20] Chiesi M Longoni S Limbruno U Cardiac alpha-crystallin. III. Involvement during heart ischemia. Mol Cell Biochem 1990 97 129 36 2280761 10.1007/BF00221054

[21] Farahbakhsh ZT Huang QL Ding LL Altenbach C Steinhoff HJ Horwitz J Hubbell WL Interaction of alpha-crystallin with spin-labeled peptides. Biochemistry 1995 34 509 16 7819243 10.1021/bi00002a015

[22] Treweek TM Rekas A Lindner RA Walker MJ Aquilina JA Robinson CV Horwitz J Perng MD Quinlan RA Carver JA R120G alpha B-crystallin promotes the unfolding of reduced alpha-lactalbumin and is inherently unstable. FEBS J 2005 272 711 24 15670152 10.1111/j.1742-4658.2004.04507.x

[23] Ecroyd H Meehan S Horwitz J Aquilina JA Benesch JL Robinson CV Macphee CE Carver JA Mimicking phosphorylation of alphaB-crystallin affects its chaperone activity. Biochem J 2007 401 129 41 16928191 10.1042/BJ20060981 PMC1698675

[24] Ganea E Harding JJ Molecular chaperones protect against glycation-induced inactivation of glucose-6-phosphate dehydrogenase. Eur J Biochem 1995 231 181 5 7628468

[25] Blakytny R Harding JJ Prevention of the fructation-induced inactivation of glutathione reductase by bovine alpha-crystallin acting as a molecular chaperone. Ophthalmic Res 1996 28 19 22 8727959 10.1159/000267938

[26] Heath MM Rixon KC Harding JJ Glycation-induced inactivation of malate dehydrogenase protection by aspirin and a lens molecular chaperone, [alpha]-crystallin. Biochim Biophys Acta 1996 1315 176 84 8611656 10.1016/0925-4439(95)00120-4

[27] Haley DA Horwitz J Stewart PL Image restrained modeling of alphaB-crystallin. Exp Eye Res 1999 68 133 6 9986751 10.1006/exer.1998.0610

[28] Carver JA Aquilina JA Truscott RJ Ralston GB Identification by 1H NMR spectroscopy of flexible C-terminal extensions in bovine lens alpha-crystallin. FEBS Lett 1992 311 143 9 1397302 10.1016/0014-5793(92)81386-z

[29] Carver JA Probing the structure and interactions of crystallin proteins by NMR spectroscopy. Prog Retin Eye Res 1999 18 431 62 10217479 10.1016/s1350-9462(98)00027-5

[30] Regini JW Grossmann JG Burgio MR Malik NS Koretz JF Hodson SA Elliott GF Structural changes in alpha-crystallin and whole eye lens during heating, observed by low-angle X-ray diffraction. J Mol Biol 2004 336 1185 94 15037078 10.1016/S0022-2836(03)00814-3

[31] Regini JW Grossmann JG Timmins P Harding JJ Quantock AJ Hodson SA Elliott GF X-ray- and neutron-scattering studies of alpha-crystallin and evidence that the target protein sits in the fenestrations of the alpha-crystallin shell. Invest Ophthalmol Vis Sci 2007 48 2695 700 17525201 10.1167/iovs.06-0559

[32] Burgio MR Kim CJ Dow CC Koretz JF Correlation between the chaperone-like activity and aggregate size of alpha-crystallin with increasing temperature. Biochem Biophys Res Commun 2000 268 426 32 10679221 10.1006/bbrc.1999.2036

[33] Clarke MJ Artero JB Moulin M Callow P Carver JA Griffiths PC Haertlein M Harding JJ Meek KM Timmins P Regini JW Investigation of [gamma]E-crystallin target protein binding to bovine lens alpha-crystallin by small-angle neutron scattering. Biochim Biophys Acta 2010 1800 392 7 20004233 10.1016/j.bbagen.2009.12.001

[34] Lindner RA Kapur A Carver JA The interaction of the molecular chaperone, alpha-crystallin, with molten globule states of bovine alpha-lactalbumin. J Biol Chem 1997 272 27722 9 9346914 10.1074/jbc.272.44.27722

[35] Lindner RA Treweek TM Carver JA The molecular chaperone alpha-crystallin is in kinetic competition with aggregation to stabilize a monomeric molten-globule form of alpha-lactalbumin. Biochem J 2001 354 79 87 11171082 10.1042/0264-6021:3540079 PMC1221631

[36] Carver JA Lindner RA Lyon C Canet D Hernandez H Dobson CM Redfield C The interaction of the molecular chaperone alpha-crystallin with unfolding alpha-lactalbumin: a structural and kinetic spectroscopic study. J Mol Biol 2002 318 815 27 12054825 10.1016/S0022-2836(02)00144-4

[37] Goward CR Nicholls DJ Malate-dehydrogenase - a model for structure, evolution and catalysis. Protein Sci 1994 3 1883 8 7849603 10.1002/pro.5560031027 PMC2142602

[38] Horwitz J Huang QL Ding LL The native oligomeric organization of alpha-crystallin, is it necessary for its chaperone function? Exp Eye Res 2004 79 817 21 15642318 10.1016/j.exer.2004.05.007

[39] Horwitz J Huang QL Ding L Bova MP Lens alpha-crystallin: chaperone-like properties. Methods Enzymol 1998 290 365 83 9534176 10.1016/s0076-6879(98)90032-5

[40] Konarev PV Volkov VV Sokolova AV Koch MHJ Svergun DI PRIMUS: a Windows PC-based system for small-angle scattering data analysis. J Appl Crystallogr 2003 36 1277 82

[41] Guinier A, Fournet F. Small Angle Scattering of X-Rays. New York: Wiley Interscience; 1955.

[42] Burchard W Static and dynamic light-scattering from branched polymers and bio-polymers. Adv Polym Sci 1983 48 1 124

[43] Bova MP Yaron O Huang Q Ding L Haley DA Stewart PL Horwitz J Mutation R120G in alphaB-crystallin, which is linked to a desmin-related myopathy, results in an irregular structure and defective chaperone-like function. Proc Natl Acad Sci USA 1999 96 6137 42 10339554 10.1073/pnas.96.11.6137 PMC26848

[44] Bettelheim FA Ansari R Cheng QF Zigler JS Jr The mode of chaperoning of dithiothreitol-denatured alpha-lactalbumin by alpha-crystallin. Biochem Biophys Res Commun 1999 261 292 7 10425180 10.1006/bbrc.1999.1031

[45] Uversky VN Segel DJ Doniach S Fink AL Association-induced folding of globular proteins. Proc Natl Acad Sci USA 1998 95 5480 3 9576907 10.1073/pnas.95.10.5480 PMC20402

[46] Kim KK Kim R Kim SH Crystal structure of a small heat-shock protein. Nature 1998 394 595 9 9707123 10.1038/29106

[47] Stamler R Kappe G Boelens W Slingsby C Wrapping the alpha-crystallin domain fold in a chaperone assembly. J Mol Biol 2005 353 68 79 16165157 10.1016/j.jmb.2005.08.025

[48] Bagnéris C Bateman OA Naylor CE Cronin N Boelens WC Keep NH Slingsby C Crystal Structures of alpha-Crystallin Domain Dimers of alpha B-Crystallin and Hsp20. J Mol Biol 2009 392 1242 52 19646995 10.1016/j.jmb.2009.07.069

[49] Haley DA Horwitz J Stewart PL The small heat-shock protein, alphaB-crystallin, has a variable quaternary structure. J Mol Biol 1998 277 27 35 9514758 10.1006/jmbi.1997.1611

[50] Peschek J Braun N Franzmann TM Georgalis Y Haslbeck M Weinkauf S Buchner J The eye lens chaperone alpha-crystallin forms defined globular assemblies. Proc Natl Acad Sci USA 2009 106 13272 7 19651604 10.1073/pnas.0902651106 PMC2726422

[51] Carver JA Aquilina JA Truscott RJ A possible chaperone-like quaternary structure for alpha-crystallin. Exp Eye Res 1994 59 231 4 7835412 10.1006/exer.1994.1101

[52] Bettelheim FA Kinetics of chaperoning of dithiothreitol-denatured alpha-lactalbumin by alpha-crystallin. Int J Biol Macromol 2002 30 161 9 12063118 10.1016/s0141-8130(02)00014-4

[53] Haley DA Bova MP Huang QL McHaourab HS Stewart PL Small heat-shock protein structures reveal a continuum from symmetric to variable assemblies. J Mol Biol 2000 298 261 72 10764595 10.1006/jmbi.2000.3657

[54] Skouri-Panet F Quevillon-Cheruel S Michiel M Tardieu A Finet S. sHSPs under temperature and pressure: the opposite behaviour of lens alpha-crystallins and yeast HSP26. Biochim Biophys Acta 2006 1764 372 83 16476575 10.1016/j.bbapap.2005.12.011

[55] Spinozzi F Mariani P Rustichelli F Amenitsch H Bennardini F Mura GM Coi A Ganadu ML Temperature dependence of chaperone-like activity and oligomeric state of alpha B-crystallin. Biochim Biophys Acta 2006 1764 677 87 16581320 10.1016/j.bbapap.2006.02.003

[56] Augusteyn RC alpha-Crystallin polymers and polymerization: the view from down under. Int J Biol Macromol 1998 22 253 62 9650080 10.1016/s0141-8130(98)00023-3

[57] Thurn A Burchard W Niki R Structure of Casein Micelles. 2. Alpha-S1-Casein. Colloid Polym Sci 1987 265 897 902

[58] Haslbeck M Franzmann T Weinfurtner D Buchner J Some like it hot: the structure and function of small heat-shock proteins. Nat Struct Mol Biol 2005 12 842 6 16205709 10.1038/nsmb993

[59] Ecroyd H Carver JA The effect of small molecules in modulating the chaperone activity of alphaB-crystallin against ordered and disordered protein aggregation. FEBS J 2008 275 935 47 18218039 10.1111/j.1742-4658.2008.06257.x

[60] Treweek TM Rekas A Walker MJ Carver JA A quantitative NMR spectroscopic examination of the flexibility of the C-terminal extensions of the molecular chaperones, αA- and αB-crystallin. Exp Eye Res 2010 91 691 9 20732317 10.1016/j.exer.2010.08.015

[61] Stengel F Baldwin AJ Painter AJ Jaya N Basha E Kay LE Vierling E Robinson CV Benesch JLP Quaternary dynamics and plasticity underlie small heat shock protein chaperone function. Proc Natl Acad Sci USA 2010 107 2007 12 20133845 10.1073/pnas.0910126107 PMC2836621

[62] Laganowsky A Benesch JLP Landau M Ding LL Sawaya MR Cascio D Huang QL Robinson CV Horwitz J Eisenberg D Crystal structures of truncated alphaA and alphaB crystallins reveal structural mechanisms of polydispersity important for eye lens function. Protein Sci 2010 19 1031 43 20440841 10.1002/pro.380 PMC2868245

[63] Lindner RA Kapur A Mariani M Titmuss SJ Carver JA Structural alterations of alpha-crystallin during its chaperone action. Eur J Biochem 1998 258 170 83 9851707 10.1046/j.1432-1327.1998.2580170.x

[64] Rekas A Jankova L Thorn DC Cappai R Carver JA Monitoring the prevention of amyloid fibril formation by alpha-crystallin. FEBS J 2007 274 6290 304 18005258 10.1111/j.1742-4658.2007.06144.x

